# Gut Microbiota and Phenotypic Changes Induced by Ablation of Liver- and Intestinal-Type Fatty Acid-Binding Proteins

**DOI:** 10.3390/nu14091762

**Published:** 2022-04-22

**Authors:** Guojun Wu, Hiba R. Tawfeeq, Atreju I. Lackey, Yinxiu Zhou, Zoe Sifnakis, Sophia M. Zacharisen, Heli Xu, Justine M. Doran, Harini Sampath, Liping Zhao, Yan Y. Lam, Judith Storch

**Affiliations:** 1 New Jersey Institute for Food, Nutrition and Health, Rutgers University, New Brunswick, NJ 08901, USA; gary.guojun.wu@rutgers.edu (G.W.); harini.sampath@rutgers.edu (H.S.); liping.zhao@rutgers.edu (L.Z.); 2 Department of Biochemistry and Microbiology, Rutgers University, New Brunswick, NJ 08901, USA; 3 Department of Nutritional Sciences, Rutgers University, New Brunswick, NJ 08901, USA; hrt11@scarletmail.rutgers.edu (H.R.T.); atrejulackey@gmail.com (A.I.L.); yinzhou@sebs.rutgers.edu (Y.Z.); zoesifnakis@gmail.com (Z.S.); sophia.zacharisen@gmail.com (S.M.Z.); heli0115@hotmail.com (H.X.); justine5522@gmail.com (J.M.D.); 4 Rutgers Center for Lipid Research, Rutgers University, New Brunswick, NJ 08901, USA; 5 Gut Microbiota and Metabolism Group, Centre for Chinese Herbal Medicine Drug Development, School of Chinese Medicine, Hong Kong Baptist University, Hong Kong, China

**Keywords:** intestinal fatty acid-binding protein, liver fatty acid-binding protein, gut microbiota

## Abstract

Intestinal fatty acid-binding protein (IFABP; FABP2) and liver fatty acid-binding protein (LFABP; FABP1) are small intracellular lipid-binding proteins. Deficiency of either of these proteins in mice leads to differential changes in intestinal lipid transport and metabolism, and to markedly divergent changes in whole-body energy homeostasis. The gut microbiota has been reported to play a pivotal role in metabolic process in the host and can be affected by host genetic factors. Here, we examined the phenotypes of wild-type (WT), LFABP^−/−^, and IFABP^−/−^ mice before and after high-fat diet (HFD) feeding and applied 16S rRNA gene V4 sequencing to explore guild-level changes in the gut microbiota and their associations with the phenotypes. The results show that, compared with WT and IFABP^−/−^ mice, LFABP^−/−^ mice gained more weight, had longer intestinal transit time, less fecal output, and more guilds containing bacteria associated with obesity, such as members in family *Desulfovibrionaceae*. By contrast, IFABP^−/−^ mice gained the least weight, had the shortest intestinal transit time, the most fecal output, and the highest abundance of potentially beneficial guilds such as those including members from *Akkermansia*, *Lactobacillus*, and *Bifidobacterium*. Twelve out of the eighteen genotype-related bacterial guilds were associated with body weight. Interestingly, compared with WT mice, the levels of short-chain fatty acids in feces were significantly higher in LFABP^−/−^ and IFABP^−/−^ mice under both diets. Collectively, these studies show that the ablation of LFABP or IFABP induced marked changes in the gut microbiota, and these were associated with HFD-induced phenotypic changes in these mice.

## 1. Introduction

Fatty acid-binding proteins (FABPs) are a family of 14–15 kDa intracellular proteins that are thought to transport fatty acids (FAs) and other lipophilic molecules within the cell interior [1,2]. Liver fatty acid-binding protein (LFABP, FABP1), the first member identified [3,4], is highly expressed in the liver and also found abundantly in the proximal small intestine [5]. In contrast to other FABPs, which bind a single molecule of ligand, LFABP binds two molecules of long-chain fatty acids (LCFAs) [6,7] or two molecules of monoacylglycerol [8], in addition to a variety of other hydrophobic ligands including cholesterol, bile acids [9], lysophospholipids [10], and endocannabinoids [11,12]. In addition to LFABP, intestinal fatty acid-binding protein (IFABP, FABP2) [5,13] is also found in the small intestine (SI), its sole tissue of expression. IFABP has a high affinity for both saturated and unsaturated LCFAs with a single ligand-binding site [6,14,15], and has recently been shown to bind endocannabinoids as well [12]. While their precise functions are not entirely known, both of the enterocyte FABPs are considered to be important as reservoirs for cytoplasmic FAs, preventing lipotoxicity caused by elevated intracellular free fatty acid (FFA) levels, and to traffic FAs to enzymes involved in their synthetic incorporation into triglycerides (TGs) and phospholipids (PLs), or in their oxidation [2,16]. It is also suggested that FABPs may traffic their ligands to proteins involved in cellular signaling [17].

Although both IFABP and LFABP are expressed in the same cell type, the proximal intestinal enterocyte, and while both bind LCFAs, we have demonstrated that the two proteins are functionally distinct. In vitro studies revealed markedly different mechanisms of ligand transfer between IFABP or LFABP and membranes [2,18]. Further, it was found using null mice for either of these genes that LFABP is involved in directing intestinal monoglycerides (MGs) toward TG synthesis and FAs to oxidative pathways, while IFABP directs FAs toward synthesis of TG [8,19]. In addition to these local cellular effects, many phenotypic and metabolic differences at the whole-body level have also been observed between the LFABP and IFABP null mice. Specifically, null mice for LFABP become heavier and fatter on a high-fat diet (HFD) than WT mice, with a lower respiratory exchange ratio (RER), indicative of increased fat oxidation [19,20,21], supporting a role of LFABP in regulating whole-body energy homeostasis. The increase in body weight of LFABP^−/−^ mice was, in part, due to higher food intake, which may be secondary to the increase in mucosal levels of the endocannabinoids (ECs) 2-arachidonoylglycerol (2-AG) and arachidonoylethanolamine (AEA) [19]. Despite their obese phenotype, LFABP^−/−^ mice are normoinsulinemic, display higher levels of spontaneous activity than the WT control mice [19], and are protected against the HFD-induced decline in endurance-exercise capacity [22]. Due to these aforementioned metabolic changes, null mice for LFABP are considered an example of a metabolically healthy obese “MHO” phenotype.

Conversely to LFABP^−/−^, we found that ablation of IFABP results in less weight gain upon HFD feeding relative to WT, with IFABP^−/−^ mice having a higher RER, indicative of greater carbohydrate oxidation, and a lower food intake than WT mice [19]. IFABP ablation did not result in higher fecal lipid concentration [19]. However, we recently found that HFD-fed IFABP^−/−^ mice have blunt villi, a thinner muscularis layer, reduced goblet cell and Paneth cell densities, reduced transit time, increased fecal excretion, and increased intestinal permeability [23]. These findings may indicate nutrient malabsorption, including lipid malabsorption, which likely contributes to the leaner phenotype observed in IFABP null mice [23]. The markedly different whole-body phenotypes in LFABP^−/−^ vs. IFABP^−/−^ mice support a growing understanding of gut lipid metabolism and transport as an important regulatory factor in whole-body energy homeostasis. Notably, the phenotypic changes were not due to compensatory changes in the expression of the other FABPs located in the small intestine (SI) of IFABP^−/−^ and LFABP^−/−^ mice [19], further supporting the independent and distinct roles of the proximal SI FABPs, IFABP, and LFABP in intestinal and whole-body homeostasis.

It is now well established that gut microbiota plays an essential role in host health and can modulate many host metabolic processes including lipid metabolism [24] and energy homeostasis [25] through multiple direct and indirect biological mechanisms. These include production of a variety of bioactive compounds such as short-chain fatty acids (SCFAs), lipopolysaccharide (LPS), secondary bile acids, and others [25,26,27,28]. The structure of the gut microbiota is dynamic and can be affected by the amount and composition of dietary carbohydrates and fats [29,30,31,32]. While most of the products of dietary lipid digestion are absorbed in the proximal small intestine, a minority will pass through the gastrointestinal tract and directly modulate the gut microbiota composition in the distal intestine, via modulation of bacterial growth and by influencing bacterial metabolism as substrates [26]. Additionally, host genetic factors can affect the gut microbiota composition. For example, using 113 different strains in the Hybrid Mouse Diversity Panel, Org et al. found that 7 host loci were significantly associated with the gut microbiota composition [33]. The genes in the identified loci were involved in processes related to lipid metabolism, innate immune responses, and acute-phase immunological responses to lipopolysaccharides [33].

To gain insight into whether the observed dramatic whole-body phenotypic divergence between IFABP^−/−^ and LFABP^−/−^ mice was associated with the gut microbiota, here we compared the microbiome of WT, IFABP^−/−^, and LFABP^−/−^ mice before and after an 11-week high-saturated-fat feeding period. Our findings indicate that alterations in bacterial communities as a function of genotype and secondary to HFD feeding are associated with the lean phenotype of the IFABP^−/−^ mice, and with the MHO phenotype of the LFABP^−/−^ mice.

## 2. Materials and Methods

### 2.1. Animals and Diets

LFABP^−/−^ mice on a C57BL/6N background were generously provided by Binas and coworkers [34]. The mice were additionally backcrossed with C57BL/6J mice from The Jackson Laboratory (Bar Harbor, ME) as described previously [8,22]. IFABP^−/−^ mice used in the present studies were a substrain bred by intercrossing of the original IFABP^−/−^ mice [13], and were also on a C57BL/6J background as described [8,19]. WT C57BL/6J mice from The Jackson Laboratory bred in our facility were used as controls. Six mice were in each genotypic group. Mice were maintained on a 12 h light/dark cycle and allowed *ad libitum* access to standard rodent chow (Purina Laboratory Rodent Diet 5015). At 2 months of age, male LFABP^−/−^, IFABP^−/−^, and WT mice were housed 2–3 per cage and fed a 45% kcal fat HFD (D10080402; Research Diets, Inc., New Brunswick, NJ, USA) for 11 weeks; the lipid sources were cocoa butter (43% kcal) and soybean oil (2% kcal) (Table 1).

### 2.2. Body Weight and Food Intake

At 2 months of age, mice were fed the HFD. The mice were maintained on this diet for 11 weeks, and body weights were measured each week. Food intake was assessed using the Oxymax system (Columbus Instruments, Columbus, OH, USA) during weeks 10–11 of the feeding protocol. Mice were placed in chambers (1 mouse per chamber) with food for 48 h. The first 24 h were used as an acclimation period, while the second 24 h period was used to measure food intake.

### 2.3. Intestinal Transit Time

Transit time measurements were performed on non-fasted mice between weeks 10 and 11 of the HFD period. Prior to the start of the experiment, mice were individually housed. After two hours of acclimation, mice were given 250 μL of 6% carmine red and 0.5% methylcellulose (Sigma-Aldrich, St. Louis, MO, USA) in PBS by oral gavage. After gavaging the mice, the cages were checked every 10 min and the time of appearance of the first red fecal pellet recorded [35,36].

### 2.4. Total Fecal Excretion

Mice were housed 2–3 per cage. Feces from each cage were collected every 3–4 days between weeks 10 and 11 of the HFD feeding period, dried overnight at 60 °C, and then weighed. The weight of the feces was divided by the number of mice in the cage, and by the number of days of collection. In order to control for differences in food intake, fecal excretion in grams was normalized by dividing it by the respective 24 h food intake.

### 2.5. Gut Microbiota Analyses

Fresh fecal pellets were collected from 6 individual mice per genotype at baseline and again after 11 weeks of HFD feeding. Samples were snap-frozen in liquid nitrogen and stored at −80 °C until analysis. Genomic DNA was extracted using the QIAmp Power Fecal DNA kit (QIAGEN, Germantown, MD, USA), as per manufacturer instructions. The hypervariable region V4 of the 16S rRNA gene was amplified using the 515F and 806R primers modified by Parada et al. [37] and Apprill et al. [38] and sequenced using the Ion GeneStudio S5 (ThermoFisher Scientific, Waltham, MA, USA). Primers were trimmed from the raw reads using Cutadapt [39] in QIIME 2 [40]. Amplicon sequence variants (ASVs) [41] were obtained by denoising using the dada2 denoise-single command in QIIME 2 with parameters --p-trim-left 0 --p-trunc-len 215. Spurious ASVs were further removed by abundance filtering [42]. A phylogenetic tree of ASVs was built using the QIIME 2 commands alignment mafft, alignment mask, phylogeny fastree, and phylogeny midpoint-root to generate weighted UniFrac metrics. Taxonomy assignment was performed using the q2-feature-classifier plugin [43] in QIIME 2 based on the silva database (release 132) [44]. The data were rarified to 17,000 reads/sample for subsequent analyses.

Overall gut microbiota structure was evaluated using alpha diversity indices (Shannon index and observed ASVs) and beta diversity distance metric (weighted UniFrac). Principal coordinates analysis (PCoA) was performed using the R “ape” package [45] to visualize differences in gut microbiota structure between treatment groups along principal coordinates that accounted for most of the variations. Random Forest analysis was performed and cross-validated using the R “randomForest” package [46] and the “rfcv” function, respectively, to test for correlations between gut microbiota composition and body weight. Figures were visualized by the R “ggplot2” package [47] and “pheatmap” package [48].

ASV shared by >25% of the samples were considered prevalent and selected for the guild-level analysis. Pairwise correlations among the ASVs were calculated using the method described by Bland and Altman [49]. The correlation values were converted to a correlation distance (1 − correlation value) and the ASVs were clustered using the Ward clustering algorithm. From the top of the clustering tree, permutational multivariate analysis of variance (PERMANOVA; 9999 permutations with a *p* < 0.001 cut-off) was used to sequentially determine whether the two clades were significantly different and accordingly clustered the prevalent ASVs into guilds [50].

### 2.6. Statistical Analysis

Body weight, body weight change, transit time, and fecal output were analyzed using one-way ANOVA with Tukey’s post hoc between groups and repeated-measures ANOVA with Tukey’s post hoc between time points. Shannon index, ASV number, and distance to WT mice were analyzed using a Mann–Whitney test between groups and Wilcoxon matched-pairs signed-ranks test between time points. At each time point, differential guilds between the groups were identified by using a Kruskal–Wallis test with Dunn’s post hoc. A Random Forest (RF) regression model with leave-one-out cross-validation was used to regress body weight on the guild abundance by using R randomForest packages. All statistical analyses were performed using GraphPad Prism (version 9.0.1 for Mac, GraphPad Software, San Diego, CA, USA) and R version 4.1.1.

### 2.7. GC/MS Analysis of SCFAs in Fecal Samples

SCFA species, including acetate, propionate, isobutyrate, butyrate, isovalerate, and valerate from fecal samples of WT, IFABP^−/−^, and LFABP^−/−^ mice were analyzed by GC/MS as described previously [51], at the core facility of the New Jersey Institute for Food, Nutrition, and Health of Rutgers University.

## 3. Results

### 3.1. Body Weight Gain Differs in WT, IFABP^−/−^, and LFABP^−/−^ Mice after Chronic HF Feeding

**Figure 1:**
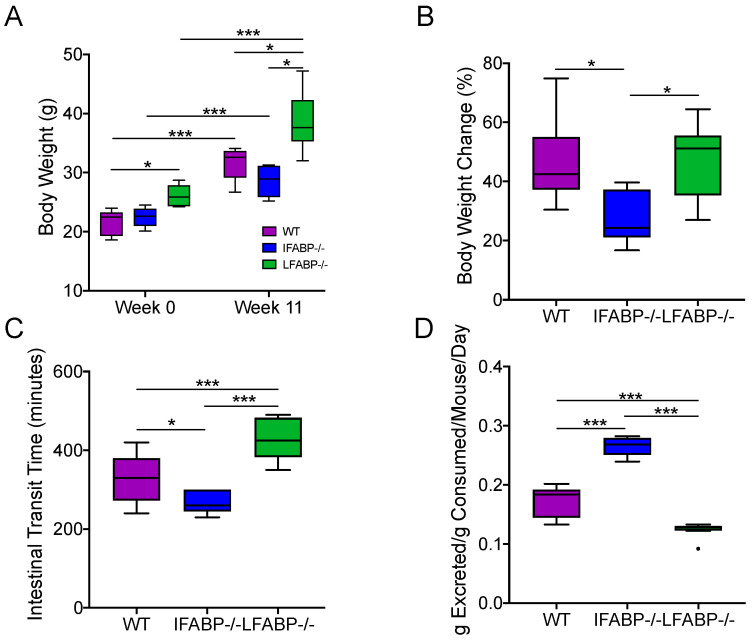
Effect of IFABP and LFABP knockout on body weight (**A**), body weight change (**B**), intestinal transit time (**C**), and total fecal output (**D**). Repeated-measures ANOVA with Tukey’s post hoc was applied in (**A**). One-way ANOVA with Tukey’s post hoc was applied in (**B**–**D**). * *p* < 0.05, *** *p* < 0.001. (**B**,**C**) were from a separate group of mice with the same genotypes and fed the same HFD. *N* = 6 for each group. LFABP; FABP1: Intestinal fatty acid-binding protein (IFABP; FABP2) and liver fatty acid-binding protein.

After 11 weeks of 45% kcal HF feeding, IFABP^−/−^ mice gained less weight and remained lean when compared to both WT and LFABP^−/−^ mice, in agreement with previous results (Figure 1A,B) [19,23]. Compared with WT mice, LFABP^−/−^ mice had a significantly higher body weight at both week 0 and week 11 and had a similar body weight change (%) after the 11 weeks of HFD (Figure 1A,B).

### 3.2. Intestinal Transit Time and Total Fecal Excretion Are Altered in Mice Lacking IFABP and LFABP

As we have reported [23], IFABP^−/−^ mice displayed faster intestinal transit time on the HFD, and higher fecal output normalized for food intake, suggesting some malabsorption of lipid and other nutrients (Figure 1C,D). Interestingly, the 45% kcal HF-fed LFABP^−/−^ mice displayed significantly slower intestinal transit times than their WT controls (Figure 1C), and a significant decrease in total fecal excretion normalized for total food intake (Figure 1D). These changes may contribute, in part, to the increased body weight gain relative to the WT [19]. The observed changes in the intestinal transit time and fecal excretion in both IFABP^−/−^ and LFABP^−/−^ mice, relative to WT and to each other, prompted us to examine potential differential impacts of these genetic modifications on the gut microbiota [52].

### 3.3. The Microbiota Composition Is Altered by IFABP and LFABP Ablation and Shows Different Responses to HFD

**Figure 2:**
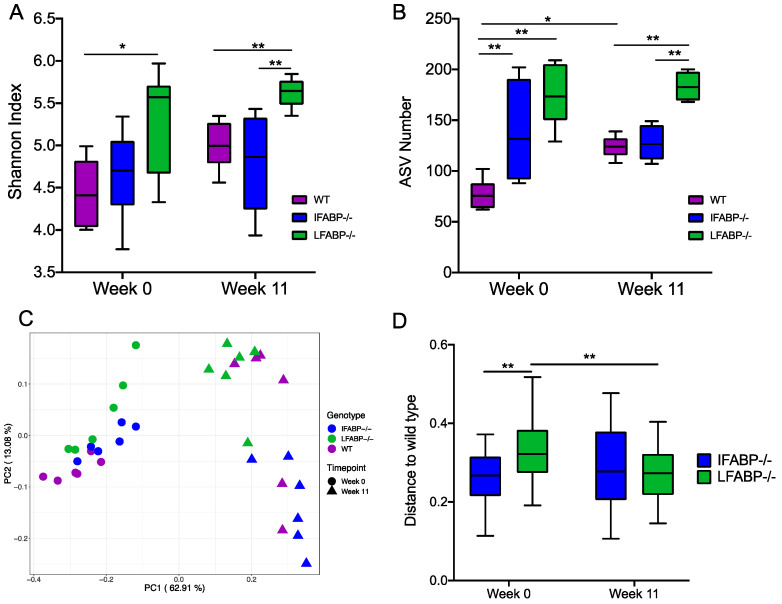
Effect of IFABP and LFABP knockout and a HF diet on the gut microbiota. (**A**) Shannon Index; (**B**) ASV number; (**C**) Principal coordinate plot based on weighted UniFrac distance; (**D**) Weighted UniFrac distance from IFABP^−/−^ and LFABP^−/−^ to WT at each time point. Data at different timepoints within the same genotype group were compared using the Wilcoxon matched-pairs signed-ranks test (two-tailed) and data at the same timepoint between the groups were compared using the Mann–Whitney test (two-tailed). * *p* < 0.05, ** *p* < 0.01. Boxes show the medians and the interquartile ranges (IQRs), and the whiskers denote the lowest and highest values within 1.5 times the IQR from the 1st and 3rd quartiles. *N* = 6 for each group.

To explore whether IFABP^−/−^ and LFABP^−/−^ mice displayed alterations in the gut microbiota, we collected fecal samples from WT, IFABP^−/−^, and LFABP^−/−^ mice (*n* = 6 per group) at both week 0 (8 weeks of age, prior to the HFD feeding period) and at week 11 of HF feeding, to profile gut microbiota composition via 16S rRNA gene V4 sequencing. In total, 785 bacterial amplicon sequence variants (ASVs) [41] were identified from the 36 samples. At week 0, LFABP^−/−^ mice had a significantly higher Shannon index than WT (Figure 2A). At week 11, the differences between LFABP^−/−^ and WT mice remained and LFABP^−/−^ mice also had a significantly higher Shannon index than IFABP^−/−^ mice. Within each genotype, there was no change in Shannon index from week 0 to week 11. Both knockout groups had significantly more ASVs than WT mice at week 0 (Figure 2B). At week 11, the ASV number showed the same differential pattern as the Shannon index among the three groups. Only in WT mice was there a significant increase in ASV number from week 0 to week 11. These results show that IFABP^−/−^ mice have increased gut microbiota diversity relative to WT only under normal chow, while LFABP^−/−^ mice have increased diversity relative to WT under both normal chow and following prolonged HF feeding, and to IFABP^−/−^ mice under HFD only. In contrast to the WT mice, the HFD treatment had no effect on the alpha diversity of the gut microbiota in either of the FABP knockout mice.

To compare the overall structure of the gut microbiota across groups, scatter plots of principal coordinate analysis based on weighted UniFrac distance were constructed (Figure 2C). The HFD significantly changed the gut microbiota structure in all groups, with clear segregations observed between week 0 and week 11 within each genotype (PERMANOVA test *p* = 0.004 in each group, R^2^ = 0.75 for WT, R^2^ = 0.78 for IFABP^−/−^, R^2^ = 0.65 for LFABP^−/−^). At week 0, both knockout groups were significantly different from WT (IFABP^−/−^ vs. WT *p* = 0.009, R^2^ = 0.33; LFABP^−/−^ vs. WT *p* = 0.004, R^2^ = 0.43), while there was no significant difference between IFABP^−/−^ and LFABP^−/−^ mice (*p* = 0.072, R^2^ = 0.17). The dissimilarity between LFABP^−/−^ and WT was significantly greater than that between IFABP^−/−^ and WT (Figure 2D). After the 11-week HF feeding, the three genotypes were significantly different from each other (IFABP^−/−^ vs. WT *p* = 0.041, R^2^ = 0.29; LFABP^−/−^ vs. WT *p* = 0.028, R^2^ = 0.22; IFABP^−/−^ vs. LFABP^−/−^ *p* = 0.004, R^2^ = 0.58), and the dissimilarity between IFABP^−/−^ and WT was similar to that between LFABP^−/−^ and WT. These results show that compared with WT, both IFABP^−/−^ and LFABP^−/−^ significantly altered the overall gut microbiota structure under both normal chow and HFD treatment. In addition, the effect of HFD on the gut microbiota structure was more profound in IFABP^−/−^ and WT than in LFABP^−/−^ mice.

Bacteria in the gut ecosystem form complex interactions as functional groups rather than existing in isolation [53]. Members that exploit the same class of resources in a similar way can be considered as a guild [54], in which the guild members typically show co-abundance patterns. Thus, to identify potential guilds, we explored the co-abundance relationships among the 202 prevalent and dominant ASVs which were shared in at least 25% of the samples and accounted for ~90% of the total abundance. The 202 ASVs were grouped into 24 different guilds (Table S1).

**Figure 3:**
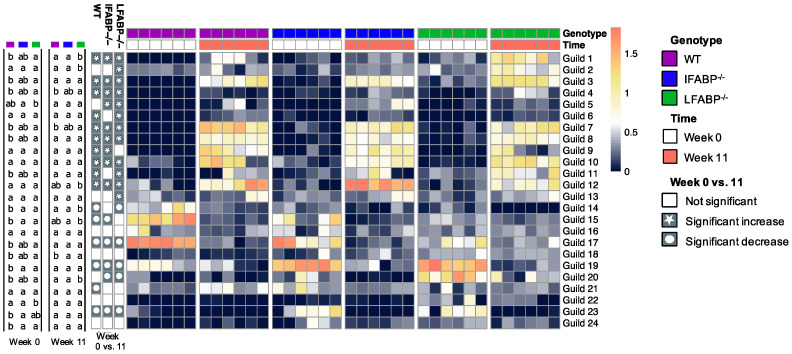
Differences and changes in the guilds of the three different genotypes. The heatmap shows the log10-transformed relative abundance of each guild. At each time point, guilds were compared among the groups using the Kruskal–Wallis test and post hoc Dunn’s test. Values not sharing common letters are significantly different from one another (*p* < 0.05). Wilcoxon matched-pairs signed-ranks test (two-tailed) was used to test the same guild between week 0 and week 11 within each genotype. *p* < 0.05 was considered as significant. *N* = 6 for each group.

As shown in Figure 3, at week 0, the abundance of three guilds (Guilds #19, 23, and 24) was significantly higher and that of two guilds (Guilds #14 and 15) was significantly lower in IFABP^−/−^ mice compared with WT. A comparison of LFABP^−/−^ and WT mice revealed even more significantly different guilds, i.e., 11 (Guilds #1, 3, 4, 7, 8, 11, 18, 19, 20, 22, and 24) were higher in abundance and 3 (Guilds #14, 15, and 17) were lower in the LFABP^−/−^ mice. Among the 24 differentially regulated guilds, Guilds #19 and 24 increased, and Guilds #14 and 15 decreased in both knockout groups. These results show that, under a low-fat chow diet, both FABP gene knockouts affected several functional guilds. IFABP^−/−^ changed fewer guilds than LFABP^−/−^, consistent with the aforementioned results that the dissimilarity between IFABP^−/−^ and WT was smaller than that between LFABP^−/−^ and WT.

Over the HFD feeding period from week 0 to week 11, 12 guilds (Guilds #1, 2, 3, 4, 5, 6, 7, 8, 10, 11, 12, and 13) increased and 5 (Guilds #14, 17, 19, 20, and 23) decreased significantly in LFABP^−/−^ mice; 9 guilds (Guilds #1, 3, 4, 5, 7, 8, 9, 10, and 12) increased and 5 (Guild #15, 17, 19, 20 and 23) decreased significantly in IFABP^−/−^ mice; 11 guilds (Guilds #1, 3, 4, 6, 7, 8, 9, 10, 11, 12, and 21) increased and 5 (Guild #14, 15, 17, 19, and 23) decreased significantly in WT mice (Figure 3). Among these 20 HF-responding guilds, 10 (Guilds #1, 3, 4, 7, 8, 10, and 12 which increased; Guilds #17, 19, and 23 which decreased) displayed changes in the same direction in all the groups, while the other 10 changed in two or only one of the groups. These results indicate that while some of the HFD-induced changes were independent of genotype, the gut microbiota of WT, IFABP^−/−^, and LFABP^−/−^ mice also displayed differential responses to the HFD.

At week 11 of the HFD, only Guild #18 showed a significant difference between IFABP^−/−^ and WT, being higher in IFABP^−/−^. Compared with WT, the LFABP^−/−^ had five guilds (Guilds #1, 2, 4, 18, and 20) that were significantly higher and two guilds (Guilds #7 and 14) that were lower. Under both normal chow and HFD, no guilds showed consistent differences between WT and IFABP^−/−^. However, Guilds #1, 4, 18, and 20 were consistently higher in LFABP^−/−^ compared with WT mice. These results indicate that, at the guild level, the differences between the two knockout groups and WT remain but become smaller after HFD feeding as the number of different guilds decreased. Notably, however, the differences in four guilds between LFABP^−/−^ and WT mice were present regardless of the diet.

### 3.4. Associations between Gut Microbiota and Body Weight

**Figure 4:**
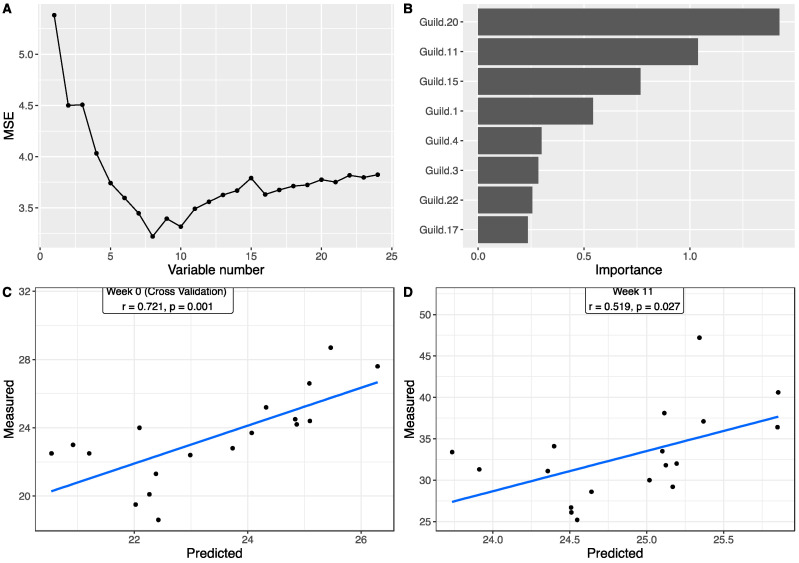
The association between the gut microbiota and body weight at week 0, prior to HF feeding (chow fed from weaning until 8 weeks of age). Random Forest (RF) model regressing body weight on the guild abundance at week 0. (**A**) shows the number of variables and mean squared error of the corresponding model. (**B**) The RF assigns a mean error rate, or feature-importance score, to each feature; this value indicates the extent to which each predictor contributes to the accuracy of the model. (**C**) Significantly positive correlation between the measured body weight and the predicted values from leave-one-out cross-validation based on RF model. (**D**) Significantly positive correlation between the measured body weight and the predicted values from guild abundance at week 11 based on the model trained in (**A**). Pearson correlation was applied.

To explore the associations between the gut microbiota and body weight, we applied a Random Forest regression model to correlate the 24 guilds and body weight. Using the data at week 0, based on the leave-one-out cross-validation and feature-selection process, the best regression model with minimum mean squared error for body weight contained eight guilds (Figure 4A,B), all of which showed differences between the three genotype groups. Each of the eight guilds also showed significant correlation with body weight based on Spearman correlations (Table S2). Particularly, among them, three guilds showed very large differences between the three genotype groups (Figure S1A). Guild #17 accounted for 47.97% of the total abundance in WT, 22.09% in IFABP^−/−^, and 5.29% in LFABP^−/−^ mice. Similarly, Guild #15 was the most abundant in WT (27.19%), followed by IFABP^−/−^ (6.82%) and LFABP^−/−^ (1.60%). In contrast, the abundance of Guild #20 was the lowest in WT (0.42%) but higher in IFABP^−/−^ (2.23%) and markedly higher in LFABP^−/−^ (18.55%). The predicted body weights from cross-validation were significantly correlated with the measured values (r = 0.721, *p* = 0.001) (Figure 4C). This result indicates that the genotype-related guilds associate with the host body weight under normal chow at 8 weeks of age.

**Figure 5:**
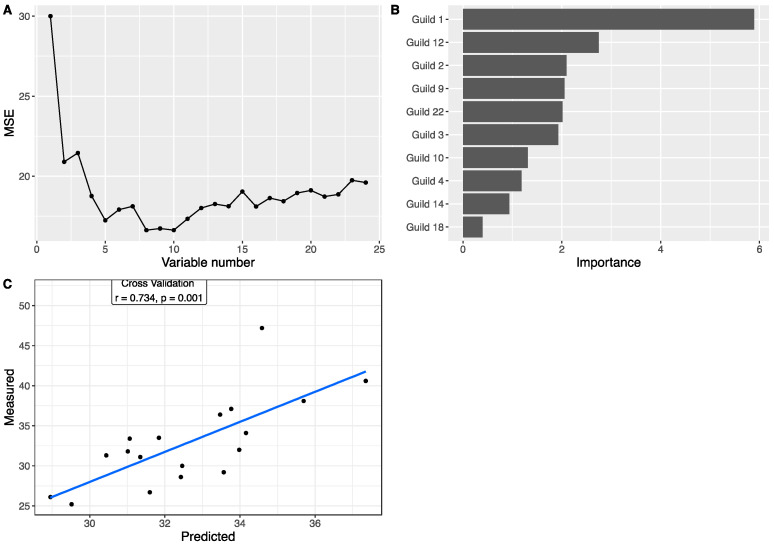
The association between the gut microbiota and body weight following 11 weeks of the HF diet. Random Forest (RF) model regressing body weight on the guild abundance at week 11. (**A**) shows the number of variables and mean squared error of the corresponding model. (**B**) The RF assigns a mean error rate, or feature-importance score, to each feature; this value indicates the extent to which each predictor contributes to the accuracy of the model. (**C**) Scatter plot of the measured body weight and the predicted values from leave-one-out cross-validation. Pearson correlation was applied.

To determine associations between guilds and body weight following the HFD period, we applied the Random Forest regression model to correlate the 24 guilds and body weight at week 11 (Figure 5A,B). Ten guilds were included in the best model with a minimum mean squared error. Individually, based on Spearman correlations, 5 out of the 10 showed significant correlation with body weight as well (Table S2). Among these 10, 5 guilds had >5% differences between the three genotypes (Figure S1B). Guild #12 was most dominant in the IFABP^−/−^ mice (20.21% in WT, 43.81% in IFABP^−/−^, and 6.23% in LFABP^−/−^). In contrast, the abundance of Guild #10 was the lowest in IFABP^−/−^ mice (13.70% in WT, 5.69% in IFABP^−/−^, and 12.41% in LFABP^−/−^). Guilds #1 and 3 were most abundant in LFABP^−/−^ mice (Guild #1: 2.15% in WT, 0.98% in IFABP^−/−^, and 11.63% in LFABP^−/−^; Guild #3: 7.53% in WT, 5.96% in IFABP^−/−^, and 13.43% in LFABP^−/−^). Guild #9 had the lowest abundance in LFABP^−/−^ mice (9.27% in WT, 8.32% in IFABP^−/−^, and 3.80% in LFABP^−/−^). As shown in Figure 5C, the predicted body weights from cross-validation were significantly correlated with the measured values (r = 0.734, *p* = 0.001). In addition, we found four common guilds (Guilds #1, 3, 4, and 22) in the two Random Forest regression models built on the data at week 0 and week 11. The predicted body weight values, which were based on the week 0 model and the week 11 guilds, were significantly correlated with the measured body weights at week 11 as well (r = 0.519, *p* = 0.0207) (Figure 4D). These results indicate that the associations between guilds and body weight identified under normal chow are retained, in part, after HFD feeding. The contribution of some guilds to the body weight, by contrast, were manifested only after HFD feeding.

### 3.5. IFABP and LFABP Ablation and HFD Feeding Alter Fecal SCFA Levels

**Figure 6:**
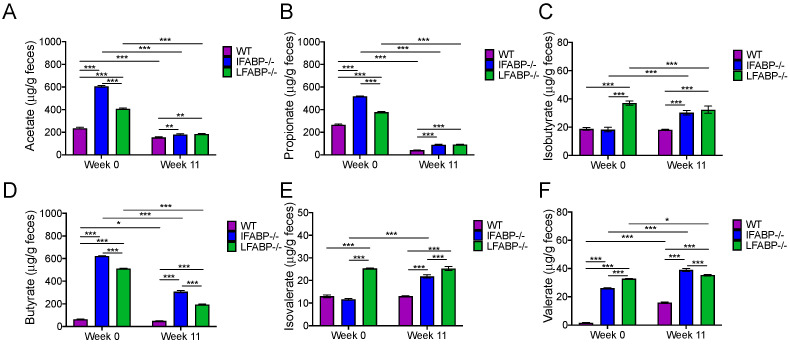
Analysis of SCFAs in WT, IFABP^−/−^, and LFABP^−/−^ mice at week 0 (chow diet from weaning until 8 weeks of age) and after 11 weeks of the HF diet. (**A**) Acetate; (**B**) Propionate; (**C**) Isobutyrate; (**D**) Butyrate; (**E**) Isovalerate; (**F**) Valerate. Feces were pooled from six mice in each genotype. Two-way ANOVA with Tukey’s post hoc was applied. * *p* < 0.05, **p < 0.01, *** *p* < 0.001. The error bars are from technical replicates.

Among the guilds associated with body weight variation, we noted that some of the members were SCFA-producing bacteria (Table S1). It has been reported that SCFAs are important gut microbial metabolites related to host energy homeostasis [25]. To explore whether the different genotypic mice had differences in SCFA levels, we measured SCFAs in the fecal samples. At week 0, prior to starting the HFD, the levels of all measured SCFAs, including acetate, propionate, isobutyrate, butyrate, isovalerate, and valerate showed significant differences between the three genotypes (Figure 6). Acetate, propionate, butyrate, and valerate were significantly higher in both IFABP^−/−^ and LFABP^−/−^ mice compared to their control counterparts (Figure 6A,B,D,F). Isobutyrate and isovalerate were significantly higher than the WT group only in the LFABP^−/−^ mice (Figure 6C,E).

After 11 weeks of HF feeding, the concentrations of SCFAs remained different between the three genotypes. In all three genotypes, acetate, propionate, and butyrate levels were significantly decreased when compared to week 0 (Figure 6A,B,D), while valerate was significantly increased after HF feeding (Figure 6F). In keeping with what was observed at week 0, all of the SCFA levels were significantly greater in both IFABP^−/−^ and LFABP^−/−^ mice when compared to the WT mice at week 11 of the HFD period. Both butyrate and valerate were higher in IFABP^−/−^ mice when compared to LFABP^−/−^ (Figure 6D,F), while isovalerate was higher in LFABP^−/−^ compared to IFABP^−/−^ mice (Figure 6E). These results indicate that differences in the levels of SCFAs are primarily due the genetic ablation of IFABP and LFABP, and persisted after chronic HF feeding.

## 4. Discussion

In the present study, we found divergent effects of IFABP vs. LFABP gene knockout on intestinal transit times and fecal output which had significant differential impact on the gut microbiota. The LFABP^−/−^ mice had significantly slower transit, i.e., longer transit times, and lower fecal output per gram consumed. In agreement with our prior findings [23], the opposite was found in the IFABP^−/−^ mice. Thus, the opposing body weight phenotypes of the IFABP and LFABP null mice are likely due, in part, to increased energy harvest in LFABP^−/−^ and decreased energy harvest in IFABP^−/−^ mice.

In recent years, it has been shown that FABPs, including both LFABP and IFABP, bind not only LCFAs, but also have high-affinity binding for the ECs 2-arachidonoylglycerol (2-AG) and anandamide [11,12,55,56]. ECs are involved in the regulation of food intake and intestinal motility through activation of the cannabinoid receptor1 (CB1R) on vagal afferent neurons [57,58,59]. It has been shown that activation of the CB1R by receptor agonists such as 2-AG inhibits peristalsis and can increase appetite [60,61,62]. Indeed, we previously showed that mucosal levels of 2-AG were lower in IFABP^−/−^ mice, whereas they were significantly higher in LFABP^−/−^ mice, when compared to their WT counterparts [19]. Thus, the highly divergent phenotypes that have been observed in body weight, in the amount of fecal output, and in the intestinal motility of both IFABP^−/−^ and LFABP^−/−^ mice could, in part, be secondary to altered CB1R activation caused by different mucosal EC levels.

Compared with WT mice, both IFABP^−/−^ and LFABP^−/−^ had altered overall gut microbiota structure under a normal chow diet. Although shifting from normal chow to HFD changed the gut microbiota structure dramatically in all the three genotypes, the responses of the gut microbiota in each genotype were different. Such differences together with their different gut microbiotas after HFD feeding may be associated with the aforementioned variations in intestinal motility. Transit time is related to bacterial composition and metabolism in the gut [52,63]. Interestingly, the LFAPB^−/−^ mice had the longest transit time and highest number of bacterial ASVs among the three genotypes, which is consistent with the finding that a long transit time associates with high microbial richness [52,63].

To assess the functional significance of the alterations in the gut microbiota, we applied guild analysis, which overcomes the pitfalls of commonly used taxonomy analysis and is a more ecologically sound approach for finding host phenotype-associated gut microbial members [50]. Under normal chow, among the eight guilds that were associated with body weight, Guilds #15, 17, and 20 showed remarkable and significant differences between the three genotypes. Guild #15 was negatively correlated with body weight and had one ASV from *Akkermansia*; the species *Akkermansia muciniphila* in this genus has been characterized as beneficial in whole-body energy metabolism [64]. Guild #17, which was negatively correlated with body weight, had ASVs from genera including *Lactobacillus* and *Lachnoclostridium*. Many members of *Lactobacillus* are considered as probiotics and are associated with host health [65], and members in *Lachnoclostridium* have been reported to be associated with an anti-obesity function [66]. Guild #20, which was positively correlated with body weight, contained one ASV from *Tyzzerella,* which have been reported as pro-inflammatory bacteria and to be related to obesity [67,68]. Compared with WT and IFABP^−/−^, LFABP^−/−^ mice not only had the lowest abundance of the potentially beneficial Guilds #15 and 17 but also highest abundance of the potentially obesogenic Guild #20.

After HF feeding, among the 10 guilds that were associated with body weight, Guild #12 was most dominant in the IFABP^−/−^ mice and negatively correlated with body weight. This guild had two ASVs from *Lactobacillus*, one from *Bifidobacterium*, one from *Ileibacterium*, one from *Lactococcus*, one from *Dubosiella newyorkensis*, two from *Lachnospiraceae*, two from *Ruminococcaceae,* one from *Enterorhabdus*, and one from *Streptococcus*. Several members in *Lactobacillus*, *Bifidobacterium*, and *Lactococcus* have been reported to attenuate HFD-induced obesity [69,70,71]. Guild #10, which had three ASVs from *Desulfovibrionaceae*, had the lowest abundance in the IFABP^−/−^ mice and positively correlated with body weight. Members of *Desulfovibrionaceae*, which produce endotoxin and hydrogen sulfide, are considered pro-inflammatory and have been reported to be positively associated with obesity and inflammation [72,73]. Guilds #1 and 3, which had ASVs from *Odoribacter*, had the highest abundance in LFABP^−/−^ mice and were positively correlated with body weight. *Odoribacter* has been reported to be positively correlated with body weight [74]. Indeed, under both diets, the LFABP^−/−^ mice had the highest body weight among the three genotypes. Overall, the LFABP^−/−^ mice had more potentially obesity-promoting guilds including bacteria such as those from *Tyzzerella*, *Desulfovibrionaceae*, and *Odoribacter,* and fewer anti-obesity guilds including bacteria from *Akkermansia*, *Lactobacillus*, *Lachnoclostridium*, and *Bifidobacterium* [64,65,66,69,70,71]. The IFABP^−/−^ mice, by contrast, had more anti-obesity and fewer obesity-promoting guilds after HFD feeding, which appears associated with their lean phenotype relative to WT and LFABP^−/−^ mice.

In addition to body weight, which was focused on here, our previous studies showed that LFABP^−/−^ mice can be considered an example of being metabolically healthy obese “MHO”, with higher levels of spontaneous activity [19] and protection against the HFD-induced decline in endurance-exercise capacity [22]. Recent human studies have highlighted that exercise can stimulate changes in the gut microbiota associated with higher SCFA production [75,76]. Thus, in addition to the different transit time noted above, higher levels of endurance activity may be considered as another factor which potentially contributes to the significant differences in the gut microbiota between WT and LFABP^−/−^ mice. Previously, we found that LFABP^−/−^ mice had higher muscle glycogen levels and an increased FA oxidation rate when compared with WT mice [22]. Here, we found that SCFAs were significantly higher in LFABP^−/−^ mice compared with WT mice. These findings are consistent with the recently proposed “gut–muscle axis” [77,78], in which SCFAs are considered as potential regulators, via increasing skeletal muscle glycogen and promoting FA uptake and oxidation [79]. Other studies have also shown that high levels of plasma and fecal acetate and propionate are associated with endurance-exercise improvement [80,81]. Thus, the gut microbiota may play an essential role in the “MHO” features of LFABP^−/−^ mice.

In previous studies, we showed that IFABP^−/−^ mice remained lean (Figure 1A), a result that was also found in the present studies, and we also showed that the IFABP^−/−^ mice had lower plasma glucose levels than their WT counterparts, and a normoinsulinimic phenotype after chronic HF feeding [19]. Here, we showed that IFABP^−/−^ mice maintain a high level of fecal SCFAs. Many studies have indicated beneficial effects of SCFAs, specifically, acetate, propionate, and butyrate, on energy homeostasis and metabolism, and their crucial role in preventing HFD-induced obesity and improving insulin sensitivity [82,83,84]. Fecal SCFA levels can be modulated by several mechanisms including colonic absorption, colonic transit time, dietary intake, and the microbiota [85]. Though a lower abundance of SCFA-producing bacteria was found in the LFABP^−/−^ mice than in the IFABP^−/−^ mice, their higher level of fecal SCFAs may be related to their longer transit time, which increases the fermentation time [86]. The higher level of fecal SCFAs in the IFABP^−/−^ mice may be related to increased SCFA production or reduced absorption. A higher abundance of SCFA-producing bacteria was identified in IFABP^−/−^ mice, which suggests the possibility of increased SCFA production. However, as IFABP^−/−^ mice have reduced transit time, this may result in reduced absorption of SCFAs. In order to dissect the contributions of the observed SCFA changes to the IFABP^−/−^ and LFABP^−/−^ mice phenotypes, the measurement of SCFA absorption will be of interest.

In summary, our results show that the gut microbiota is associated with the high-fat-diet-induced whole-body phenotypes of IFABP^−/−^ and LFABP^−/−^ mice. To determine whether the structure of the microbiota is an essential mediator of the effects of these gene knockouts on host phenotypes, future studies will assess the impact of transplanting the gut microbiota from the IFABP^−/−^ and LFABP^−/−^ mice to germ-free or antibiotic-treated WT mice. The FABP gene family includes a group of diverse proteins that have important roles in regulating host metabolism and have been shown to be related to several metabolic diseases [87]. Better understanding of their functions and mechanisms of action, which may be mediated by the gut microbiota, will facilitate FABP-related drug development and therapeutic approaches for metabolic diseases.

## Figures and Tables

**Table 1:** FA composition of high-saturated-fat diet [19].

	HFS Grams/4057 kcal
C16	49.9
C16:1	0.4
C18	64.3
C18:1	65.2
C18:2	10.7
C18:3	1.0
%	
Saturated fatty acids	60.0
Monounsaturated fatty acids	33.9
Polyunsaturated fatty acids	6.1

## Data Availability

The raw gut microbiome sequencing data have been deposited to the sequence read archive at NCBI under the BioProject ID PRJNA772007.

## Supplementary Materials

The following supporting information can be downloaded at: https://www.mdpi.com/article/10.3390/nu14091762/s1, Figure S1: The average abundance of the guilds which were included in the best model of Random Forest analysis and had >5% difference among the three genotypes; Table S1: 202 ASVs were grouped into 24 different guilds; Table S2: Spearman correlation between Guilds and body weight.
