# Supplementary material for: Gut Microbiota and Phenotypic Changes Induced by Ablation of Liver- and Intestinal-Type Fatty Acid-Binding Proteins
Source: Nutrients. 2022 Apr 22;14(9):1762. doi: 10.3390/nu14091762 (PMC9099671; doi:10.3390/nu14091762)
Supplement: Supplementary file 1 [file nutrients-14-01762-s001.zip › nutrients-1674162-supplementary.pdf]

Table S1. 202 ASVs were grouped into 24 different guilds

ASV ID	Guild ID	ASV ID	Guild ID
Odoribacter.ASV_16	Guild 1	Oscillibacter.ASV_88	Guild 8
Ruminiclostridium.9.ASV_29	Guild 1	Lachnospiraceae.NK4A136.group.ASV_104	Guild 8
Turicibacter.ASV_44	Guild 1	Lachnospiraceae.UCG.004.ASV_133	Guild 8
Ruminococcaceae.UCG.010.ASV_155	Guild 1	Lachnospiraceae.ASV_163	Guild 8
Ruminococcaceae.UCG.004.ASV_212	Guild 1	Lachnospiraceae.ASV_11	Guild 9
Ruminiclostridium.5.ASV_215	Guild 1	Acetatifactor.ASV_38	Guild 9
Ruminococcaceae.ASV_258	Guild 1	Lachnospiraceae.NK4A136.group.ASV_58	Guild 9
Eubacterium.nodatum.group.ASV_289	Guild 1	Ruminiclostridium.ASV_59	Guild 9
Alistipes.ASV_47	Guild 2	Oscillibacter.ASV_64	Guild 9
Bacteroides.ASV_56	Guild 2	Ruminiclostridium.ASV_118	Guild 9
Odoribacter.ASV_84	Guild 2	Ruminiclostridium.5.ASV_132	Guild 9
Alistipes.ASV_86	Guild 2	Lachnospiraceae.ASV_191	Guild 9
Muribaculaceae.ASV_94	Guild 2	Desulfovibrionaceae.ASV_2	Guild 10
Muribaculaceae.ASV_105	Guild 2	Brachyspira.sp..NSH.25.ASV_100	Guild 10
Muribaculaceae.ASV_119	Guild 2	Desulfovibrionaceae.ASV_101	Guild 10
Muribaculaceae.ASV_161	Guild 2	Ruminococcaceae.ASV_157	Guild 10
Lachnospiraceae.ASV_206	Guild 2	Lachnospiraceae.ASV_203	Guild 10
Muribaculaceae.ASV_208	Guild 2	Desulfovibrio.ASV_286	Guild 10
Ruminococcaceae.ASV_292	Guild 2	Lachnospiraceae.ASV_331	Guild 10
Lachnospiraceae.bacterium.609.ASV_13	Guild 3	Muribaculaceae.ASV_12	Guild 11
Lachnoclostridium.ASV_23	Guild 3	Faecalibaculum.ASV_48	Guild 11
Odoribacter.ASV_36	Guild 3	Muribaculaceae.ASV_95	Guild 11
Rikenellaceae.RC9.gut.group.ASV_37	Guild 3	Rikenellaceae.RC9.gut.group.ASV_103	Guild 11
Lachnospiraceae.ASV_55	Guild 3	Parabacteroides.ASV_123	Guild 11
Rikenellaceae.RC9.gut.group.ASV_62	Guild 3	Roseburia.ASV_199	Guild 11
Lachnospiraceae.ASV_130	Guild 3	Clostridium.sp..Culture.Jar.19.ASV_274	Guild 11
Anaerovorax.ASV_174	Guild 3	Ileibacterium.ASV_3	Guild 12
Blautia.ASV_188	Guild 3	Lactobacillus.ASV_4	Guild 12
Mollicutes.RF39.ASV_340	Guild 3	Bifidobacterium.ASV_5	Guild 12
Oscillibacter.ASV_352	Guild 3	Lactobacillus.ASV_45	Guild 12
Ruminococcaceae.ASV_374	Guild 3	Lactococcus.ASV_46	Guild 12
Romboutsia.ASV_114	Guild 4	Dubosiella.newyorkensis.ASV_66	Guild 12

Mollicutes.RF39.ASV_116	Guild 4	Ruminococcaceae.ASV_76	Guild 12
Eubacterium.coprostanoligenes.group.ASV_171	Guild 4	Ruminococcaceae.UCG.003.ASV_168	Guild 12
Acetatifactor.ASV_173	Guild 4	Clostridium.scindens.ASV_177	Guild 12
Lachnospiraceae.ASV_238	Guild 4	Lachnospiraceae.ASV_195	Guild 12
Lachnospiraceae.ASV_246	Guild 4	Enterorhabdus.ASV_364	Guild 12
Lachnospiraceae.ASV_300	Guild 4	Streptococcus.ASV_432	Guild 12
Lachnospiraceae.ASV_303	Guild 4	Bacteroides.ASV_41	Guild 13
Ruminiclostridium.ASV_57	Guild 5	Bacteroides.ASV_61	Guild 13
Oscillibacter.ASV_99	Guild 5	Lachnoclostridium.ASV_107	Guild 13
Lachnospiraceae.ASV_108	Guild 5	Peptococcaceae.ASV_137	Guild 13
Intestinimonas.ASV_134	Guild 6	Erysipelatoclostridium.ASV_141	Guild 13
Clostridium.sp..Culture.54.ASV_181	Guild 6	Lachnospiraceae.ASV_164	Guild 13
Lachnospiraceae.UCG.006.ASV_183	Guild 6	Bilophila.ASV_175	Guild 13
Ruminococcaceae.ASV_235	Guild 6	Peptococcus.ASV_185	Guild 13
Lachnospiraceae.ASV_290	Guild 6	Coriobacteriaceae.UCG.002.ASV_293	Guild 13
Ruminiclostridium.9.ASV_7	Guild 7	Rikenellaceae.RC9.gut.group.ASV_51	Guild 14
Lachnospiraceae.NK4A136.group.ASV_8	Guild 7	Bacteroides.ASV_79	Guild 14
Ruminiclostridium.ASV_21	Guild 7	Muribaculaceae.ASV_83	Guild 14
Lachnospiraceae.ASV_54	Guild 7	Gastranaerophilales.ASV_126	Guild 14
Lachnospiraceae.ASV_65	Guild 7	Gastranaerophilales.ASV_152	Guild 14
Roseburia.ASV_110	Guild 7	Muribaculaceae.ASV_156	Guild 14
Blattella.germanica..German.cockroach..ASV_150	Guild 7	Akkermansia.ASV_10	Guild 15
Clostridium.sp..Culture.1.ASV_31	Guild 8	Parabacteroides.distasonis.ASV_18	Guild 15
Lachnospiraceae.ASV_33	Guild 8	Muribaculaceae.ASV_20	Guild 15
Lachnospiraceae.ASV_69	Guild 8	Muribaculaceae.ASV_75	Guild 15
Tyzzerella.ASV_71	Guild 8	Muribaculaceae.ASV_93	Guild 15

ASV ID	Guild ID	ASV ID	Guild ID
Muribaculaceae.ASV_129	Guild 15	Muribaculaceae.ASV_159	Guild 19
Muribaculaceae.ASV_136	Guild 15	Muribaculaceae.ASV_265	Guild 19
Lachnoclostridium.ASV_146	Guild 15	Helicobacter.ASV_15	Guild 20
Bacteroides.ASV_149	Guild 15	Mucispirillum.ASV_24	Guild 20
Alistipes.ASV_176	Guild 15	Muribaculaceae.ASV_28	Guild 20
Ureaplasma.ASV_317	Guild 15	Muribaculaceae.ASV_63	Guild 20
Muribaculaceae.ASV_39	Guild 16	Lachnospiraceae.ASV_70	Guild 20
Helicobacter.ASV_42	Guild 16	Tyzzerella.3.ASV_77	Guild 20
Mucispirillum.ASV_82	Guild 16	Muribaculaceae.ASV_201	Guild 20
Muribaculaceae.ASV_109	Guild 16	Lachnospiraceae.NK4A136.group.ASV_282	Guild 20
Lachnoclostridium.ASV_6	Guild 17	Ruminococcaceae.ASV_315	Guild 20
Muribaculaceae.ASV_9	Guild 17	Helicobacter.ASV_35	Guild 21
Muribaculaceae.ASV_14	Guild 17	Lachnospiraceae.NK4A136.group.ASV_72	Guild 21
Bacteroides.ASV_19	Guild 17	Ruminiclostridium.9.ASV_87	Guild 21
Muribaculaceae.ASV_26	Guild 17	Lachnospiraceae.ASV_131	Guild 21
Lactobacillus.ASV_49	Guild 17	Desulfovibrio.ASV_142	Guild 21
Parasutterella.ASV_73	Guild 17	Eubacterium.xylanophilum.group.ASV_151	Guild 21
Muribaculaceae.ASV_80	Guild 17	Lachnospiraceae.ASV_166	Guild 21
Parabacteroides.ASV_81	Guild 17	Lachnospiraceae.ASV_189	Guild 21
Muribaculaceae.ASV_90	Guild 17	Alistipes.ASV_106	Guild 22
Rhodospirillales.ASV_153	Guild 17	Peptococcaceae.ASV_167	Guild 22
Burkholderiales.bacterium.YL45.ASV_165	Guild 17	Lachnospiraceae.ASV_172	Guild 22
Clostridium.sp..K4410.MGS.306.ASV_209	Guild 17	Oscillibacter.ASV_180	Guild 22
Muribaculaceae.ASV_222	Guild 17	Lachnospiraceae.ASV_192	Guild 22
Alistipes.ASV_316	Guild 17	Prevotellaceae.UCG.001.ASV_22	Guild 23
Lactobacillus.ASV_50	Guild 18	Muribaculaceae.ASV_85	Guild 23
Muribaculaceae.ASV_74	Guild 18	Gastranaerophilales.ASV_139	Guild 23
Muribaculaceae.ASV_135	Guild 18	Muribaculaceae.ASV_211	Guild 23

Muribaculaceae.ASV_147	Guild 18	Rhodospirillales.ASV_255	Guild 23
Muribaculaceae.ASV_160	Guild 18	Ruminococcus.1.ASV_321	Guild 23
Alistipes.ASV_299	Guild 18	Muribaculaceae.ASV_325	Guild 23
Muribaculaceae.ASV_1	Guild 19	Gastranaerophilales.ASV_379	Guild 23
Muribaculaceae.ASV_17	Guild 19	Muribaculaceae.ASV_115	Guild 24
Muribaculaceae.ASV_25	Guild 19	Ruminiclostridium.9.ASV_121	Guild 24
Muribaculaceae.ASV_30	Guild 19	Ruminiclostridium.9.ASV_138	Guild 24
Muribaculum.ASV_32	Guild 19	Lachnospiraceae.ASV_154	Guild 24
Muribaculaceae.ASV_34	Guild 19	Lachnospiraceae.ASV_179	Guild 24
Muribaculaceae.ASV_40	Guild 19	Alistipes.ASV_198	Guild 24
Muribaculaceae.ASV_43	Guild 19	Ruminiclostridium.5.ASV_252	Guild 24
Muribaculaceae.ASV_52	Guild 19	Butyricicoccus.ASV_270	Guild 24
Muribaculaceae.ASV_98	Guild 19	Lachnospiraceae.ASV_338	Guild 24

Table S2. Spearman correlation between Guilds and body weight. Guilds in the best random forest model are highlighted in bold

Week 0			Week 11		
ID	Spearman R	Spearman P	ID	Spearman R	Spearman P
**Guild1**	0.818414515	3.30E-05	**Guild1**	0.839009288	0
Guild2	0.084667022	0.738361457	**Guild2**	0.662880832	0.002715482

**Guild3**	0.736843167	0.000486399	**Guild3**	0.230134159	0.356732466
**Guild4**	0.665159485	0.002593241	**Guild4**	0.481405216	0.043100687
Guild5	0.611873151	0.006961811	Guild5	-0.305627299	0.21744367
Guild6	0.320709687	0.194434401	Guild6	-0.019607843	0.94099952
Guild7	0.610974468	0.00706838	Guild7	0.104231166	0.68037718
Guild8	0.66957182	0.002369395	Guild8	0.304437564	0.218759819
Guild9	0.50382383	0.033024292	**Guild9**	-0.273477812	0.271106988
Guild10	-0.313016529	0.205960107	**Guild10**	0.657378741	0.00384122
**Guild11**	0.773120917	0.000168285	Guild11	0.040268462	0.873950211
Guild12	0.081095041	0.749056184	**Guild12**	-0.758513932	0.000401645
Guild13	-0.003099174	0.990262336	Guild13	0.003095975	0.993438879
Guild14	-0.572018662	0.013121998	**Guild14**	-0.400619568	0.099451802
**Guild15**	-0.719669688	0.000759574	Guild15	0.596692073	0.008946653
Guild16	-0.496644361	0.036029281	Guild16	0.230134159	0.356732466
**Guild17**	-0.569953612	0.013531883	Guild17	0.457172343	0.058181902
Guild18	0.541042922	0.020415384	**Guild18**	0.244582043	0.326595445
Guild19	0.462571048	0.053246408	Guild19	-0.116615067	0.644362238
**Guild20**	0.72081741	0.000738014	Guild20	0.503677019	0.033083736
Guild21	-0.053691282	0.832426066	Guild21	0.120805385	0.633010618
**Guild22**	0.757818518	0.000269135	**Guild22**	0.464183465	0.052313421
Guild23	0.505937083	0.032177729	Guild23	0.259904321	0.297618119
Guild24	0.618020762	0.006267149	Guild24	0.221878225	0.374660368

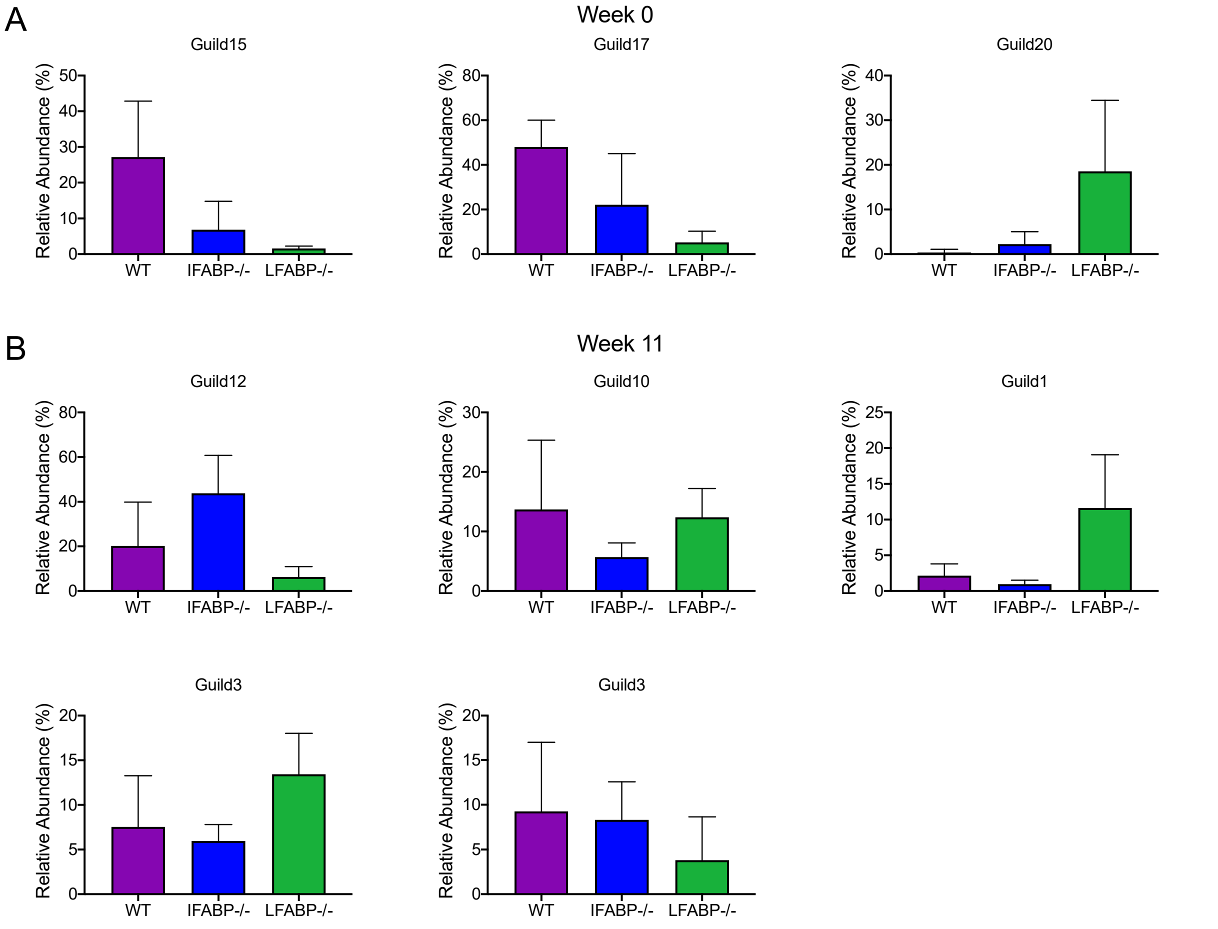

Figure S1. The average abundance of the guilds, which were included in the best model of Random Forest analysis and had > 5% difference among the three genotypes. (A) The average abundance of the guilds at week 0 prior to HF feeding initiation. (B) The average abundance of the guilds after 11 weeks of HF feeding. The bar plot shows the mean and s.d.
